# Rubredoxin 1 promotes the proper folding of D1 and is not required for heme *b*_559_ assembly in *Chlamydomonas* photosystem II

**DOI:** 10.1016/j.jbc.2023.102968

**Published:** 2023-02-02

**Authors:** Robert H. Calderon, Catherine de Vitry, Francis-André Wollman, Krishna K. Niyogi

**Affiliations:** 1 Department of Plant and Microbial Biology, University of California, Berkeley, California, USA; 2 Umeå Plant Science Centre, Department of Plant Physiology, Umeå University, Umeå, Sweden; 3 Institut de Biologie Physico-Chimique, Unité Mixte de Recherche 7141, Centre National de la Recherche Scientifique and Sorbonne Université, Institut de Biologie Physico-Chimique, Paris, France; 4 Molecular Biophysics and Integrated Bioimaging Division, Lawrence Berkeley National Laboratory, Berkeley, California, USA; 5 Howard Hughes Medical Institute, University of California, Berkeley, California, USA

**Keywords:** rubredoxin, photosystem, photosystem II, heme, photosynthesis, metalloprotein, iron, FtsH, protease, cytochrome, β-DM, β-D-maltoside, DCMU, 3-(3,4-dichlorophenyl)-1,1-dimethylurea, PSII, photosystem II, TAP, Tris acetate-phosphate

## Abstract

Photosystem II (PSII), the water:plastoquinone oxidoreductase of oxygenic photosynthesis, contains a heme *b*_559_ iron whose axial ligands are provided by histidine residues from the α (PsbE) and β (PsbF) subunits. PSII assembly depends on accessory proteins that facilitate the step-wise association of its protein and pigment components into a functional complex, a process that is challenging to study due to the low accumulation of assembly intermediates. Here, we examined the putative role of the iron[1Fe-0S]-containing protein rubredoxin 1 (RBD1) as an assembly factor for cytochrome *b*_*559*_, using the RBD1-lacking *2pac* mutant from *Chlamydomonas reinhardtii*, in which the accumulation of PSII was rescued by the inactivation of the thylakoid membrane FtsH protease. To this end, we constructed the double mutant *2pac ftsh1-1*, which harbored PSII dimers that sustained its photoautotrophic growth. We purified PSII from the *2pac ftsh1-1* background and found that α and β cytochrome *b*_559_ subunits are still present and coordinate heme *b*_559_ as in the WT. Interestingly, immunoblot analysis of dark- and low light–grown *2pac ftsh1-1* showed the accumulation of a 23-kDa fragment of the D1 protein, a marker typically associated with structural changes resulting from photodamage of PSII. Its cleavage occurs in the vicinity of a nonheme iron which binds to PSII on its electron acceptor side. Altogether, our findings demonstrate that RBD1 is not required for heme *b*_559_ assembly and point to a role for RBD1 in promoting the proper folding of D1, possibly *via* delivery or reduction of the nonheme iron during PSII assembly.

Photosystem II (PSII) is a light-driven water:plastoquinone oxidoreductase essential for the growth of all oxygenic photoautotrophic organisms. In generating oxygen as a byproduct, it also enables the growth of most, if not all, aerobic organisms on the planet. The assembly of PSII occurs in a step-wise process that requires a variety of assembly factors, some of which are conserved from cyanobacteria to higher plants (1, 2, 3, 4, 5). In a previous publication, we isolated and characterized cyanobacterial, algal, and plant mutants lacking one such assembly factor, a highly conserved rubredoxin called RubA in cyanobacteria or RBD1 in photosynthetic eukaryotes (6). Rubredoxins are small iron-containing proteins that function in electron transfer reactions. The RubA/RBD1 protein is associated with thylakoid membranes (7) and required for the assembly of PSII in *Synechocystis* sp. strain PCC 6803, *Chlamydomonas reinhardtii*, and *Arabidopsis thaliana* (6), and it was later determined to be associated with intermediate complexes during PSII assembly rather than mature, fully assembled PSII (8, 9). In *Chlamydomonas*, RBD1 appears to have roles both in assembly of PSII intermediate complexes and in photoprotection during PSII assembly and repair (8). An Arabidopsis *rbd1* mutant was recently shown to have reduced synthesis of the D1 subunit of PSII, leading to a model in which RBD1 might be required for the full translation of the *psbA* transcript encoding D1 (10). However, further elucidation of the specific role of RBD1 has been limited by our inability to isolate and study PSII that assembles in the absence of RBD1 because of the low accumulation of PSII subunits and complexes in *rbd1* mutants.

The FtsH protease complex is a membrane-localized ATP-dependent protease found in all oxygenic photoautotrophs (11). It has a well-characterized role in the degradation of damaged PSII subunits during photoinhibitory and oxidative stresses (12), and it has also been implicated in the degradation and remodeling of cytochrome *b*_6_*f* (cyt *b*_6_*f*) complexes (13). In *Chlamydomonas*, the thylakoid membrane-anchored FtsH protease exists as a heterocomplex comprised of FtsH1 and FtsH2 isoforms (13). Strikingly, two *Chlamydomonas ccb* mutants, which normally lack cyt *b*_6_*f* complexes due to mutations in cyt *b*_6_*f* assembly factors, were found to be able to accumulate aberrant forms of cyt *b*_6_*f* in a *ftsh1*-mutant background (14).

Here, we have examined the role of the RBD1 protein in the assembly of PSII in *Chlamydomonas* by inactivating the FtsH protease in the *2pac* mutant, which lacks RBD1. We show that combining the *ftsh1-1* and *2pac* mutations permits the accumulation of a misassembled variant of PSII. We find that accumulation of this variant PSII is sufficient to allow photoautotrophic growth in the absence of RBD1, and thus we show that inactivation of FtsH can be a powerful tool for studying the role of PSII assembly factors in *Chlamydomonas*.

## Results

### The lack of PSII in *2pac* is due to the instability of PSII subunits rather than a defect in translation

**Figure 1:**
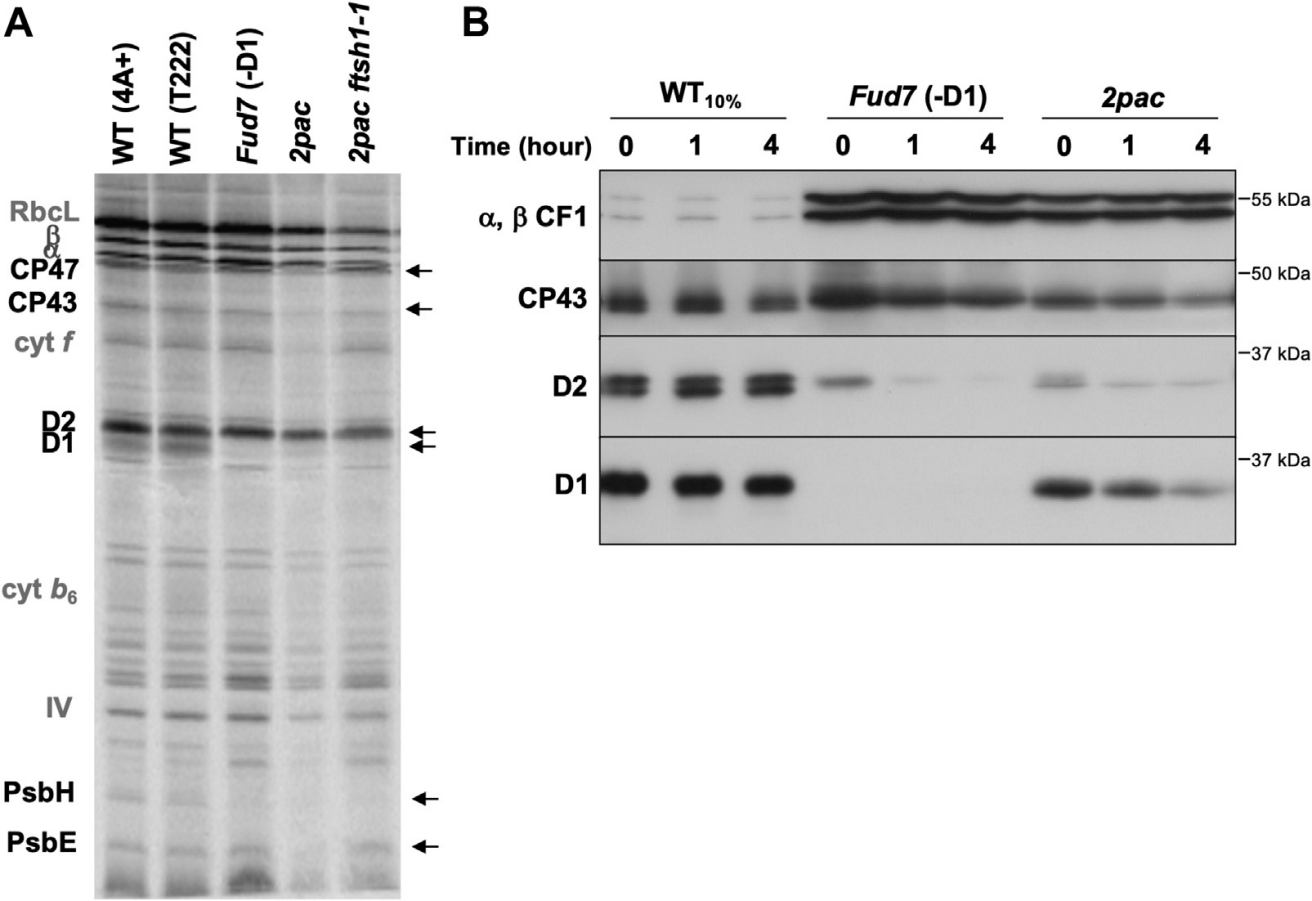
**PSII subunits are translated, but unstable in the absence of RBD1.***A*, plastid-encoded proteins from [^14^C]-acetate–radiolabeled WT (4A+ and T222), Fud7 (Δ*psbA*), *2 pac*, and *2pac ftsh1-1* whole-cell extracts separated by SDS-PAGE on a 12 to 18% polyacrylamide gel in the presence of 8 M urea and visualized by autoradiography. PSII subunits are in *black* and indicated by *arrows*. Other photosynthetic proteins are labeled in *gray*. The assignment of bands is based on mutant analysis (5, 55). *B*, immunoblot analysis of steady-state levels of α and β subunits of plastid ATP synthase (α, β CF1), D1, D2, and CP43 after 0, 1, or 4 h of incubation with chloroplast translation inhibitors. All loaded samples contained 20 μg chlorophyll, with the exception of the “WT_10%_”, which contained 2 μg chlorophyll. PSII, photosystem II.

Given that the assembly of PSII monomers occurs to a much lower extent in the *2pac* mutant than in the WT (8), we sought to investigate whether the decrease in PSII accumulation might be caused by a defect in translation of chloroplast-encoded PSII subunits. We therefore pulse-labeled *Chlamydomonas* cells for several minutes in order to detect the rates of protein translation rather than the rates of degradation. WT (4A+ or T222), *2pac*, the double mutant *2pac ftsh1-1*, and the Δ*psbA* strain Fud7 (15) cells were grown on Tris acetate-phosphate (TAP) medium (16), which contains acetate as a carbon source and allowed to reach logarithmic growth phase before being pulse-labeled with [^14^C]-acetate for 7 min in the presence of cycloheximide (to inhibit cytosolic translation). As shown in Figure 1*A*, there were no major differences in the incorporation of the radioactive signal into newly synthesized proteins between 4A+, T222, *2pac*, *2pac ftsh1-1*, and Fud7 (with the exception of D1 for Fud7). The assignment of the PSII proteins D1 (PsbA), D2 (PsbD), CP43 (PsbC), and CP47 (PsbB) is based on mutant analysis (5), and the low level of D1 labeling relative to D2 under these experimental conditions is consistent with previously published results in *Chlamydomonas* (5, 17, 18). The incorporation of radiolabeled acetate appears lower overall in *2pac* relative to both WT strains and Fud7, but PSII subunits are clearly being actively translated in *2pac* and *2pac ftsh1-1*.

Because subunits of PSII are being translated in *2pac,* we hypothesized that the lack of accumulation of PSII complexes might be due to instability of PSII subunits. To test this, WT, Fud7, and *2pac* cells were allowed to reach logarithmic growth phase before adding chloroplast translation inhibitors (lincomycin and chloramphenicol). Samples were collected at three time points (0, 1, and 4 h after incubation) for immunoblot analysis. These immunochase data showed that, over the time course, the PSII subunits D1, D2, and CP43 all rapidly disappeared in *2pac*, whereas they remained stable in WT cells (Fig. 1*B*). In the Fud7 strain, D1 was absent, and D2 and CP43 were unstable, as expected (5). In contrast to PSII proteins, the α and β subunits of the chloroplast ATP synthase were stable in all three strains (Fig. 1*B*). The data therefore suggest that genes encoding thylakoid membrane proteins are all transcribed and translated in *2pac*, and the lack of PSII is due to the specific degradation or instability of PSII subunits relative to subunits of other photosynthetic complexes.

### *2pac ftsh1-1* accumulates higher levels of PSII subunits and complexes than *2pac*

**Figure 2:**
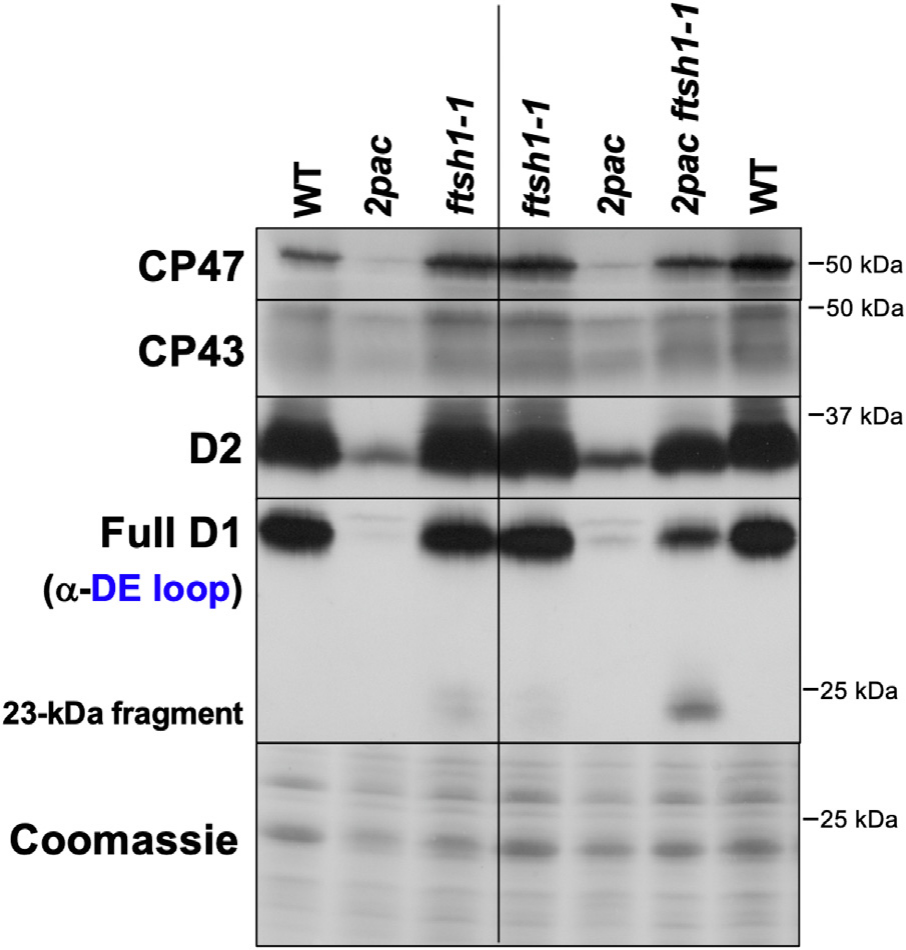
**Inactivation of FtsH increases accumulation of PSII subunits in *2pac*.** Immunoblot analysis of PSII subunit levels from a representative tetrad (tetratype tetrad 1, *right side*) obtained by crossing *2pac* and *ftsh1-1,* with parental strains and WT as controls (*left side*). Whole-cell protein extracts from the strains grown in acetate-containing TAP medium at 6 μmol photons m^−2^ s^−1^ were separated by SDS-PAGE on a 12 to 18% polyacrylamide gel in the presence of 8 M urea and immunodetected with antibodies against PSII subunits (CP47, CP43, and D2) and a peptide of the loop between the transmembrane helices D and E of D1 (D1-DE loop). Coomassie protein staining is provided as loading control. PSII, photosystem II; TAP, Tris acetate-phosphate.

**Figure S1:**
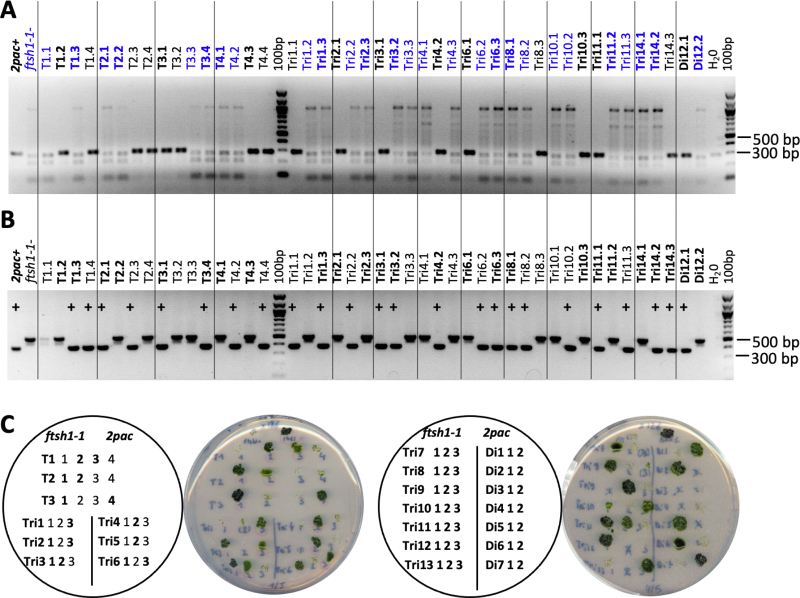
Genotyping of progeny from cross of *2pac* (mating type +) and *ftsh1-1* (mating type -) and isolation of double mutant *2pac ftsh1-1.* Cross resulted in the collection of four full tetrads (T), nine triads (Tri) and one dyad (Di). *A*, PCR analysis of *FTSH1* locus. Single band corresponds to wild-type *FTSH1* allele while three diffuse bands correspond to the *ftsh1-1* mutation. Strains in bold contain the *2pac* mutation, while those in blue contain the *ftsh1-1* mutation. *B*, PCR analysis of mating type locus. Smaller fragment corresponds to mating type +, and larger fragment corresponds to mating type -. *C*, growth of strains on TAP plates containing paromomycin (10 μg mL^−1^). Strains that grow on paromomycin correspond to those containing the *2pac* mutation (*RBD1* deletion).

**Figure S2:**
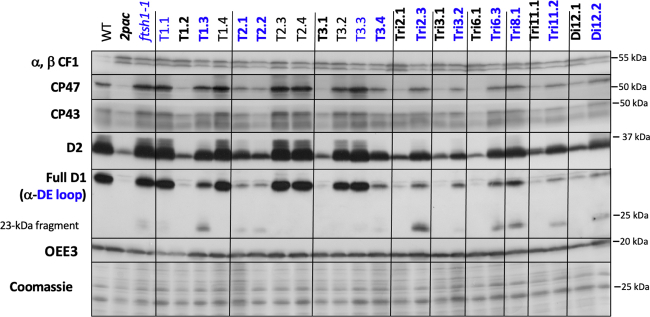
Immunoblot analysis of progeny from *2pac ftsh1-1* cross showing steady-state levels of PSII subunits. Proteins extracted from each strain were analyzed by SDS-PAGE. Strains in blue contain the *ftsh1-1* mutation, and those in bold contain the *2pac* mutation. Immunodetection of PSII proteins D1, D2, CP43, CP47, and OEE3 (PsbQ) as well as α, β subunits of plastid ATP synthase (α, β) and total protein (Coomassie). The seven strains on the left are those shown in Fig. 2 with T1.1, T1.2, T1.3 and T1.4 from this figure corresponding to the strains labeled *ftsh1-1*, *2pac, 2pac ftsh1-1* and WT, respectively, in Fig. 2.

While decreasing proteolytic activity of the FtsH protease through site-directed mutagenesis has permitted the increased accumulation of PSII subunits and subcomplexes in the cyanobacterium *Synechocystis* (19, 20, 21), the quality control systems of cyanobacteria are thought to be far less robust than in photosynthetic eukaryotes (2, 22). Thus, given the well-established role of FtsH in the degradation of damaged PSII, we hypothesized that the introduction of the *ftsh1-1* mutation into the *2pac* strain could result in a decrease in the degradation of PSII subunits and therefore an increase in steady-state levels of these proteins. We first crossed the *2pac* and *ftsh1-1* strains and isolated *2pac ftsh1-1* double mutants (Fig. S1). We then measured the accumulation of PSII subunits in these strains by immunoblot analysis after growth in low light. As shown in Figure 2, there was a marked increase in the amounts of D1, D2, CP43, and CP47 in the *2pac ftsh1-1* strain relative to both the parental *2pac* strain and the *2pac* progeny isolated from the same tetrad, although the levels of these PSII subunits were not as high as in the WT or *ftsh1-1* strains. This pattern was observed in all tested *2pac ftsh1-1* progeny (Fig. S2) with D2 levels increasing from an average of approximately 19% of WT levels in *2pac* (SD = +/− 6%) to 61% of WT levels in *2pac ftsh1-1* (SD = +/− 16%). Average CP47 levels increased from 7% of WT levels in *2pac* (SD = +/− 3%) up to 37% of WT levels in the *2pac ftsh1-1* mutant (SD = +/− 15%). In addition, immunodetection with an antibody raised against the loop connecting helices D and E of the D1 protein (α-DE loop) revealed a band at 23 kDa in *2pac ftsh1-1* and *ftsh1-1* (Fig. 2). This fragment has been detected in the single *ftsh1-1* mutant only in the light and under various stress conditions (13). It has been attributed previously to a photodamage-induced cleavage product of the full-length D1 protein (23) as well as a translational pause intermediate of D1 (24). In contrast, the WT strains did not accumulate any of the 23-kDa fragment under the tested conditions. The intensity of the 23-kDa fragment band (relative to full-length D1) was much higher in *2pac ftsh1-1* than in *ftsh1-1,* suggesting an increased PSII light sensitivity of *2pac ftsh1-1* compared to *ftsh1-1*.

**Figure 3:**
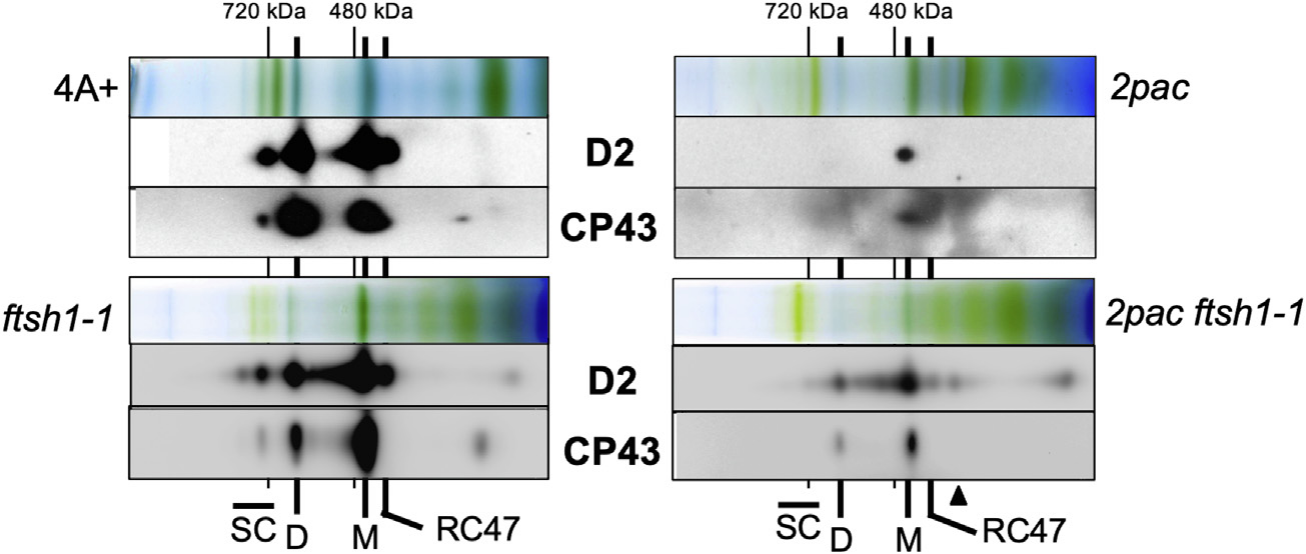
**Two-dimensional blue native (BN)/SDS-PAGE analysis of PSII in *2pac ftsh1-1* relative to parental strains.** Thylakoid membrane complexes from WT (4A+), *ftsh1-1, 2pac*, and *2pac ftsh1-1* were solubilized with 1% β-DM and separated by BN-PAGE (*top panels*), followed by SDS-PAGE in the second dimension and immunoblot analysis to detect D2 and CP43 (*lower panels*). SC, PSII super complex; D, PSII dimer; M, PSII monomer; RC47, CP43-less PSII assembly intermediate (62). Smaller complex specifically detected in *2pac ftsh1-1* with antibodies against D2 is indicated by an *arrow*. BN lanes were loaded on the basis of equal chlorophyll (7.5 μg). β-DM, β-D-maltoside; PSII, photosystem II.

To determine the assembly status of the PSII subunits in the *2pac ftsh1-1* mutant, we isolated thylakoid membranes from cells grown photoheterotrophically at 6 μmol photons m^−2^ s^−1^ and examined whether or not PSII subunits were being incorporated into complexes by 2D-PAGE analysis. In previous work with the *2pac* mutant, PSII subunits were found to assemble into PSII monomers but not dimers or supercomplexes (8). However, in the *2pac ftsh1-1* mutant, we detected these subunits in both PSII monomers and dimers (Fig. 3), indicating that these subunits were not only accumulating to greater levels (Fig. 2) but also being incorporated into higher molecular weight complexes. Additionally, in the *2pac ftsh1-1* mutant, some of the D2 subunit appeared to accumulate into a subcomplex of smaller molecular weight than the CP43-less PSII assembly intermediate RC47 complex (indicated by an arrow in Fig. 3). These smaller subcomplexes were not detected in the *ftsh1-1* mutant.

### *2pac ftsh1-1* grows photoautotrophically in low light and exhibits variable fluorescence

**Figure 4:**
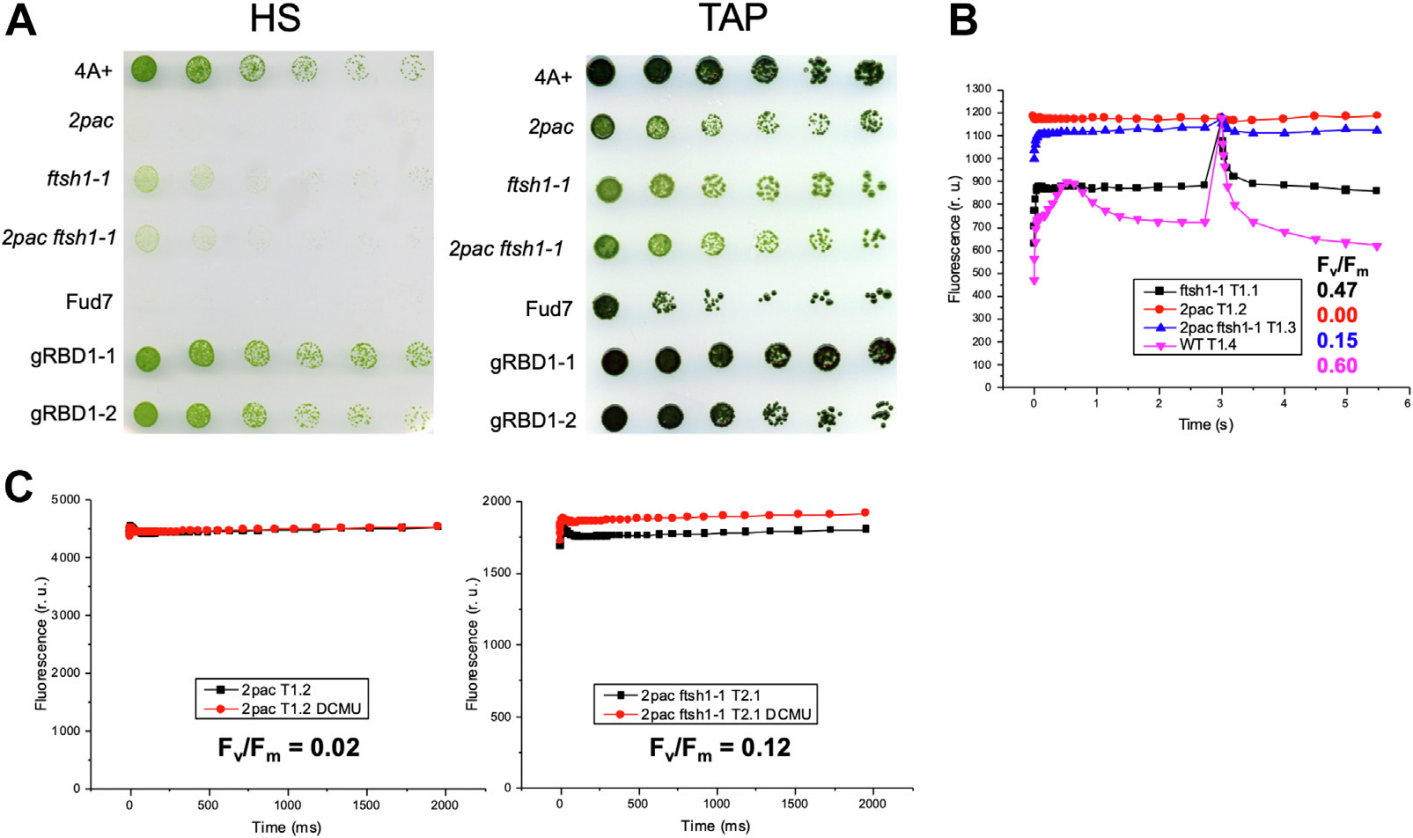
***2pac ftsh1-1* grows photoautotrophically and displays low variable fluorescence**. *A*, growth on plates without (HS, *upper panel*) and with (TAP, *bottom panel*) acetate at 30 μmol photons m^−2^ s^−1^ of WT (4A+), *2pac*, *ftsh1-1, 2pac ftsh1-1,* a strain lacking the *psbA* gene encoding the D1 protein (Fud7) and two complemented lines (gRBD1–1 and gRBD1–2). *B*, fluorescence induction kinetics in relative units (r. u.) at low actinic light of dark-adapted cells followed by a saturating pulse after 3 s to determine F_m_. Strains as in Figure 2 from tetratype tetrad 1 grown on TAP plates at 6 μmol m^−2^ s^−1^. *C*, fluorescence induction kinetics at low actinic light of dark-adapted cells in the absence (*black*) or presence (*red*) of the PSII-specific inhibitor DCMU to determine F_m_. *2pac* and *2pac ftsh1-1* strains were grown in strongly aerated TAP liquid culture at 6 μmol m^−2^ s^−1^. DCMU, 3-(3,4-dichlorophenyl)-1,1-dimethylurea; HS, high salt; PSII, photosystem II; TAP, Tris acetate-phosphate.

**Figure S3:**
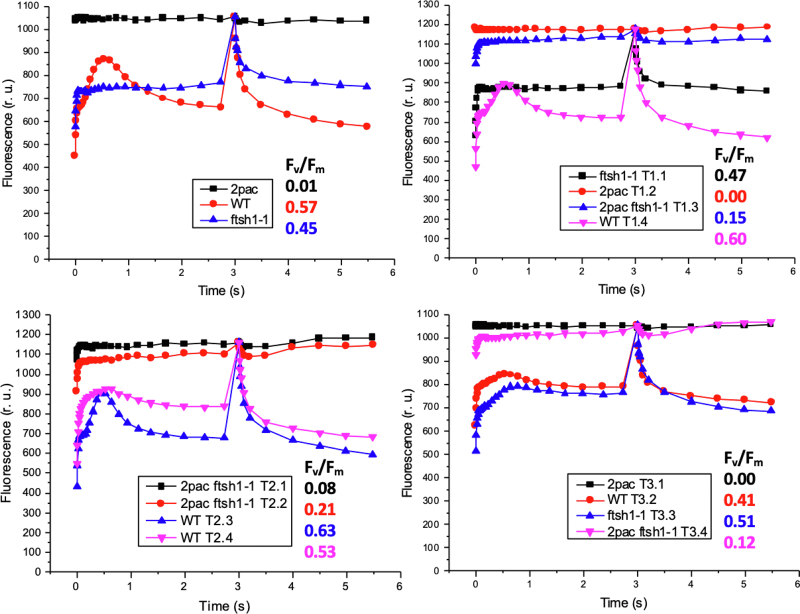
*2pac ftsh1-1* strains display some variable fluorescence. Fluorescence induction kinetics in relative units (r. u.) at low actinic light of dark-adapted cells followed by a saturating pulse to determine F_m_. Strains obtained by crossing *2pac* and *ftsh1-1*, parental and WT strains were grown on TAP plates at 6 μmol m^−2^ s^−1^. Strains shown in Fig. 4*B* are reshown here to enable direct comparison.

**Figure S4:**
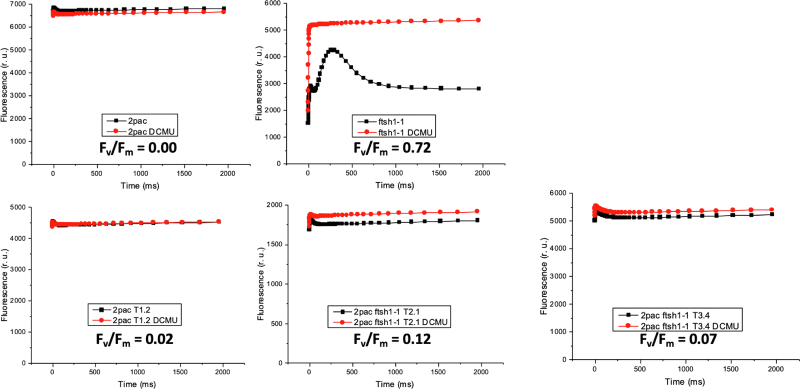
Light-grown *2pac ftsh1-1* in liquid culture displays a very low PSII activity. Fluorescence induction kinetics in relative units (r. u.) at low actinic light of dark-adapted cells in the absence (*black*) or presence (*red*) of the PSII-specific inhibitor DCMU to determine F_m_. Strains obtained by crossing mutants *2pac* and *ftsh1-1*, and parental strains were grown in strongly aerated TAP liquid culture at 6 μmol m^−2^ s^−1^. Strains shown in Fig. 4*C* are reshown here to enable direct comparison.

We next examined whether the PSII complexes that accumulate in *2pac ftsh1-1* can sustain photoautotrophic growth. As shown in Figure 4*A*, the *2pac ftsh1-1* mutant was able to grow slowly under low light conditions on high salt plates (25), a minimal medium without a carbon source, indicating that the PSII in this strain is at least partially functional. To test the activity of PSII under the same light conditions, we measured variable chlorophyll fluorescence to calculate the maximum efficiency of PSII (F_v_/F_m_) and found that *2pac ftsh1-1* exhibited low levels of variable fluorescence when grown on solid medium (Figs. 4*B* and S3) or in liquid cultures (Figs. 4*C* and S4) with F_v_/F_m_ measured in *2pac ftsh1-1* (0.12 ± 0.05), which was still lower than in *ftsh1-1* (0.55 ± 0.10) strains.

### Dark-grown *2pac ftsh1-1* displays variable fluorescence and accumulates a distinctive fragment of the D1 protein

**Figure 5:**
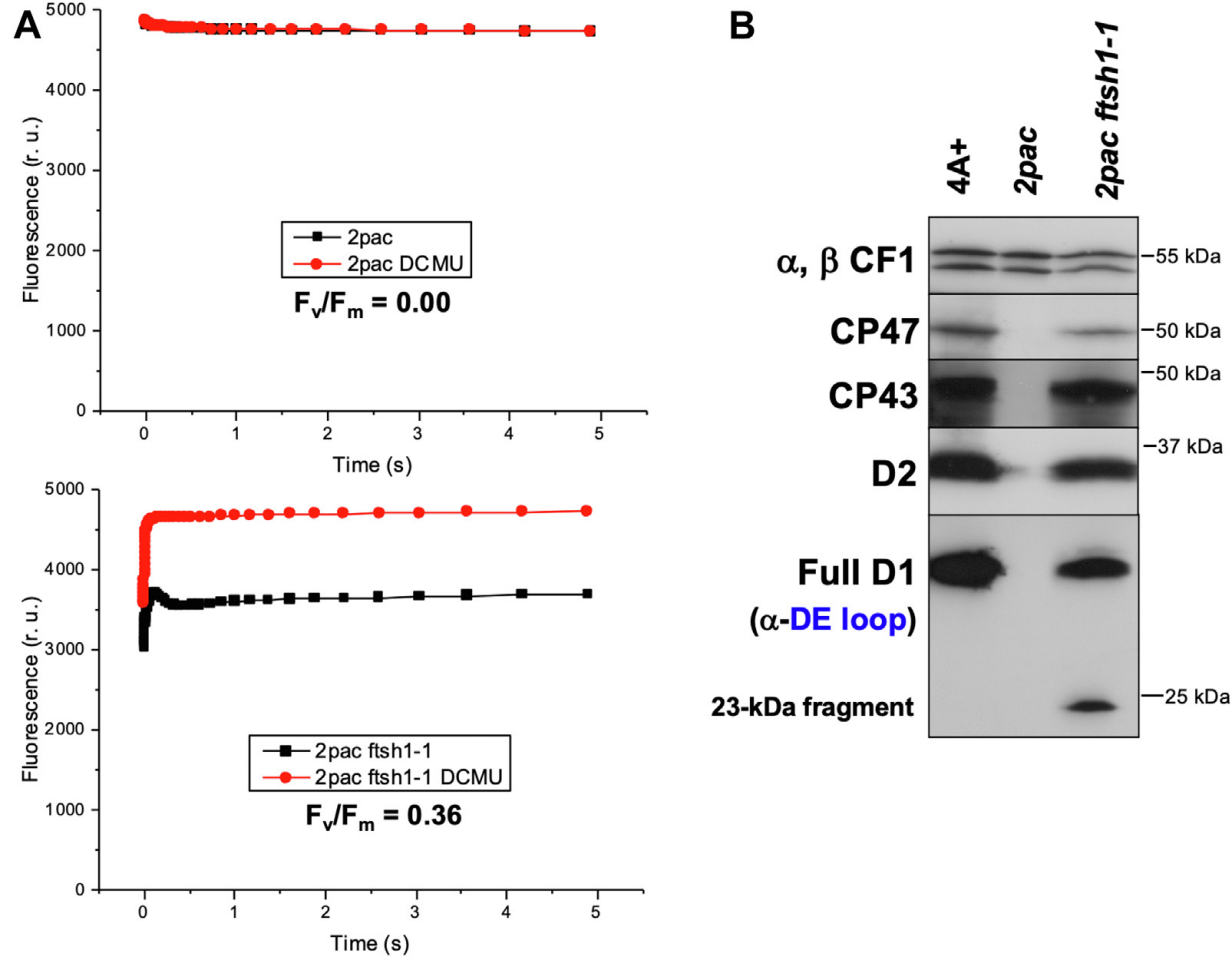
**Dark-grown *2pac ftsh1-1* displays variable fluorescence and accumulates a 23-kDa fragment of the D1 protein.***A*, fluorescence induction curves of dark-grown strongly aerated TAP liquid cultures of *2pac* (*upper panel)* and *2pac ftsh1-1* (*lower panel*) in the absence (*black*) or presence (*red*) of the PSII-specific inhibitor DCMU to determine F_m_. An absence of DCMU-altered kinetics in *2pac* indicates an absence of variable fluorescence and PSII activity (F_v_/F_m_), whereas DCMU treatment reveals a F_v_/F_m_ of 0.36 in *2pac ftsh1-1*. *B*, immunoblot analysis of PSII subunit levels from dark-grown WT (4A+), *2pac*, and *2pac ftsh1-1*. An antibody raised against a peptide from the stromal loop connecting helices D and E of the D1 protein (α-DE loop) recognizes both full-length D1 and a 23-kDa N-terminal fragment, both of which accumulate in dark-grown *2pac ftsh1-1.* DCMU, 3-(3,4-dichlorophenyl)-1,1-dimethylurea; PSII, photosystem II; TAP, Tris acetate-phosphate.

We took advantage of the ability of *Chlamydomonas* to synthesize chlorophyll in the dark as well as in the light (26) to determine whether the PSII in *2pac ftsh1-1* might be light sensitive. To address this possibility, we grew the *2pac* and *2pac ftsh1-1* mutants in darkness and again assayed F_v_/F_m_ (Fig. 5*A*). Both dark-grown and low light-grown *2pac* cells showed no variable fluorescence. The dark-grown *2pac ftsh1-1* cells displayed an appreciable increase in F_v_/F_m_ relative to low light-grown *2pac ftsh1-1* cells (0.36 vs. 0.12), consistent with higher levels of functional PSII.

We hypothesized that the low-light inactivation of PSII in *2pac ftsh1-1* might be due to either decreased abundance of PSII in the light or to a structural or conformational difference between these reaction centers and those from the WT. To test this, we first compared the accumulation of PSII subunits in dark-grown WT, *2pac*, and *2pac ftsh1-1* cells. As shown in Figure 5*B*, the accumulation of PSII subunits is comparable between dark-grown WT and *2pac ftsh1-1* cells. While overall accumulation of mature proteins was comparable, we noted that immunodetection with the α-DE loop antibody revealed a band at 23 kDa in dark-grown *2pac ftsh1-1* and not in the WT (Fig. 5*B*), as was also the case in light-grown *2pac ftsh1-1* (Fig. 2). Because this D1 fragment is absent in the dark-grown single *ftsh1-1* mutant (13) and dark-grown *2pac* (Fig. 5*B*) but present in the dark-grown double *2pac ftsh1-1* mutant (Fig. 5*B*), it arises as a consequence of combining the *2pac* mutation with inactivation of the thylakoid protease FtsH.

### Purification and characterization of PSII from dark-grown *2pac ftsh1-1* cells

We hypothesized that the presence of the 23-kDa fragment of the D1 protein in dark-grown *2pac ftsh1-1* cells might be due to a structural difference between PSII reaction centers from this strain, relative to the WT. Therefore, we purified PSII from both the *2pac ftsh1-1* strain and the WT so they could be compared. To enable purification of PSII from *2pac ftsh1-1*, we introduced a genetically encoded HIS-tag on the lumenal side of the PsbH subunit of PSII by crossing the *2pac ftsh1-1* mutant to the H-HIS strain (27). Seven full tetrads were obtained. Progeny were genotyped by PCR to determine *FTSH1* and *RBD1* alleles, and one progeny (T7A) which contained the *2pac* and *ftsh1-1* mutant alleles was selected for further analysis (Fig. S5*A*). Because *psbH* is a chloroplast-encoded gene and chloroplast inheritance is uniparental from the mating type + parent, all progeny were expected to express the HIS-tagged PsbH protein. Both the presence of PsbH-HIS and the absence of RBD1 were confirmed *via* immunoblot (Fig. S5*B*).

**Figure 6:**
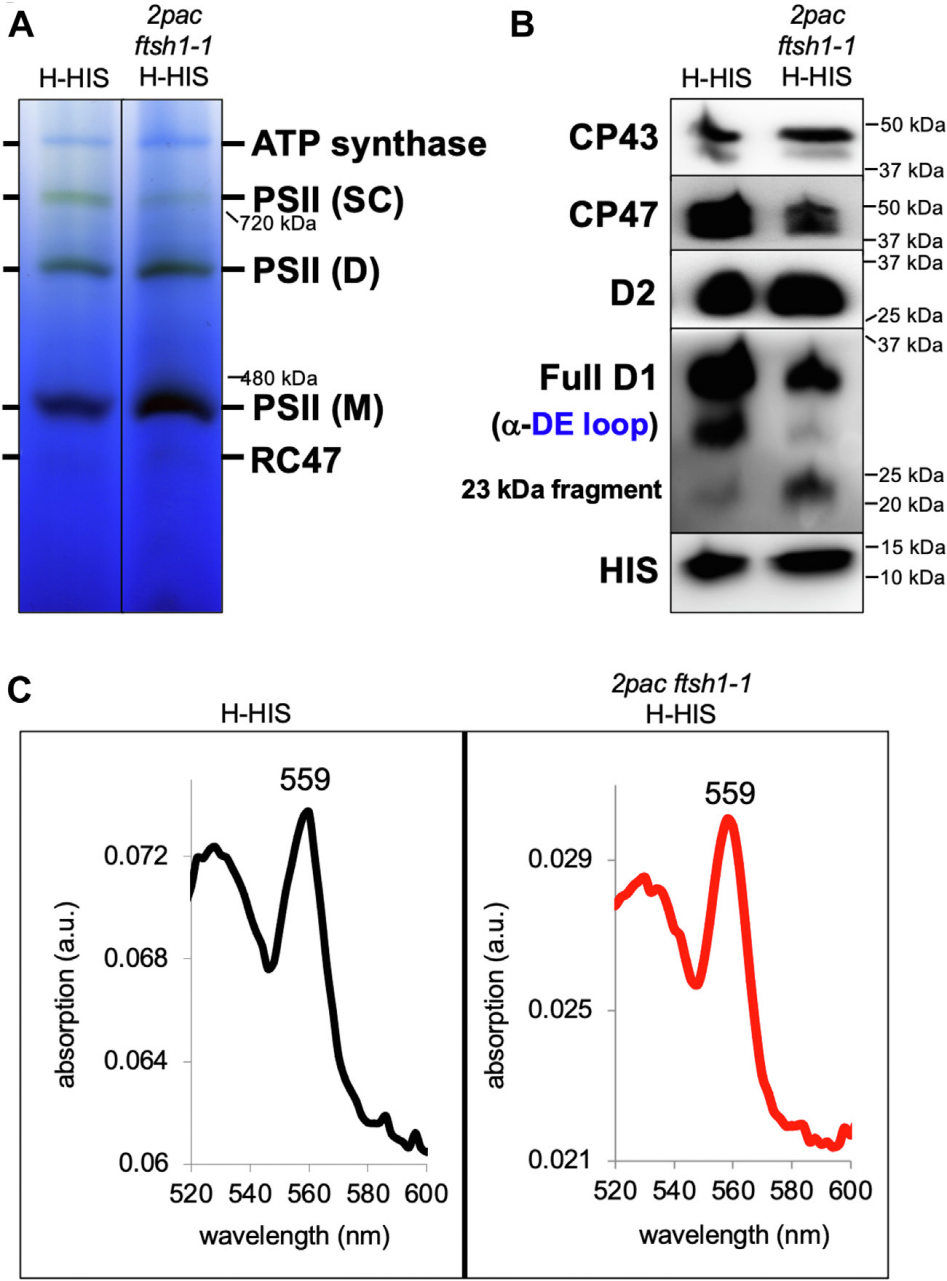
**BN-PAGE, immunoblot analysis, and absorption spectroscopy of PSII purified from WT and *2pac ftsh1-1* backgrounds.***A*, BN-PAGE analysis of Ni-affinity–purified PSII from H-HIS (strain bearing a His-tagged PsbH subunit of PSII) and *2pac ftsh1-1* H-HIS (H-HIS strain with *2pac* and *ftsh1-1* mutations introduced *via* crossing) showing the accumulation of PSII supercomplexes (SC), dimers (D), monomers (M), and RC47 subcomplexes (RC47). *B*, immunoblot analysis of excised bands corresponding to PSII monomers from both strains, showing the presence of PSII subunits. *C*, reduced minus oxidized absorption spectra of isolated PSIIs from both strains showing peaks at 559 nm, indicating the presence of the *b*-heme coordinated by cytochrome *b*_559_. PSII, photosystem II.

T7A and H-HIS were grown on acetate in darkness, and the HIS-tagged PSII complex was purified by Ni-affinity chromatography. After isolation, the PSII samples were examined *via* BN-PAGE, and PSII monomers, dimers, and supercomplexes were observed in both H-HIS and T7A. (Fig. 6*A*). As shown in Figure 6*B*, the PSII monomer from both H-HIS and T7A contained D1, D2, CP47, CP43, and HIS-tagged PsbH. To determine whether additional subunits might be lacking from the T7A dimer (or if assembly factors absent in H-HIS remained bound in T7A), the gel slices corresponding to the monomer were analyzed *via* LC-MS/MS (Table 1). The presence of D1, D2, CP47, CP43, and PsbH was confirmed as well as the additional presence of PsbO, PsbE, and PsbF in both H-HIS and T7A.

**Table 1:** PSII proteins identified *via* LC-MS/MS analysis of isolated PSII dimers from H-HIS and H-HIS *2pac ftsh1-1* (T7A)

Protein name	Uniprot No.	Gene	Protein MW (kD)	H-HIS spectral Counts	T7A spectral Counts
D1	P07753	*psbA*	39	220	398
D2	P06007	*psbD*	39	276	426
Cyt *b*559, alpha	P48268	*psbE*	9	91	70
Cyt *b*559, beta	Q08363	*psbF*	5	19	24
CP43	P56778	*psbC*	51	506	1043
CP47	P37255	*psbB*	56	427	659
PSII H subunit	P22666	*psbH*	9	31	53
PSII OEE1	P12853	*PSBO*	31	3	5

**Figure S6:**
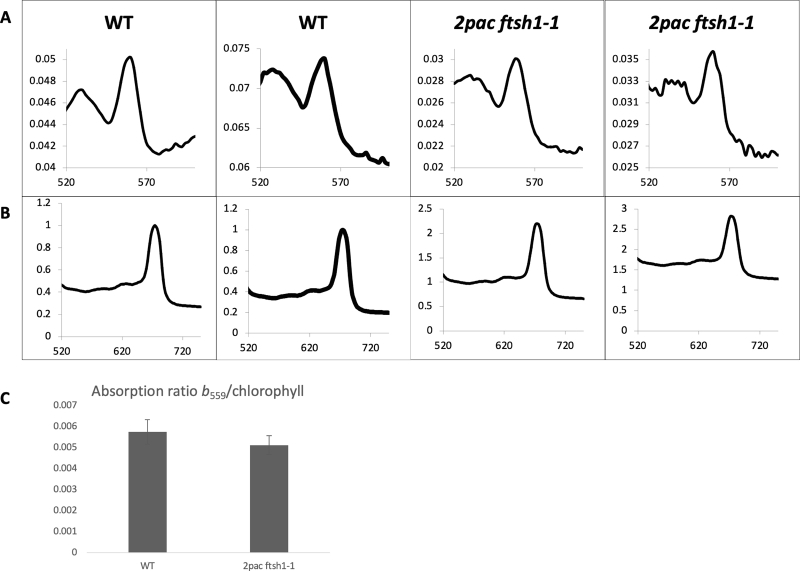
UV-Visible spectral analysis of PSII and normalization of cytochrome *b*_*559*_ signal on a per-chlorophyll basis from isolated WT and *2pac ftsh1-1* PSII. *A*, reduced minus oxidized absorption spectra of isolated PSIIs from H-HIS (WT) and T7A (*2pac ftsh1-1*) from two biological replicates showing peaks at 559 nm, indicating the presence of the *b*-heme coordinated by cytochrome *b*_559_. *B*, visible absorption spectra from 520-750 nm of isolated PSII from H-HIS (WT) and T7A (*2pac ftsh1-1*) from two biological replicates from showing absorption of chlorophylls. *C*, average intensity of peaks at 559 nm from reduced minus oxidized spectra of Ni-affinity purified PSII from H-HIS (WT) and T7A (*2pac ftsh1-1*) after normalization to chlorophyll content from the same samples. Average shown is from two biological replicates and error bars represent standard error.

Given a previously hypothesized role for RBD1 in facilitating the photoprotection of nascent PSII complexes *via* an interaction with cytochrome *b*_559_ (8), we sought to examine whether the properties of cytochrome *b*_559_ were altered in the absence of RBD1. Both subunits of cytochrome *b*_559_ (PsbE and PsbF) were detected in PSII from T7A *via* mass spectrometry (Table 1), so we also tested for the presence of the heme coordinated by these proteins by recording reduced minus oxidized UV-vis absorption spectra of isolated PSII from H-HIS and T7A. As shown in Figure 6*C*, both samples exhibited a strong peak centered at 559 nm, indicating that the PsbE and PsbF subunits detected by mass spectrometry do indeed coordinate the heme cofactor of cytochrome *b*_559_. Furthermore, after calculating the amount of heme *b*_559_ on a per-chlorophyll basis (Fig. S6), we detected no significant difference between H-HIS and T7A, consistent with the presence of WT levels of heme *b*_559_ in T7A.

## Discussion

We have previously shown that the *Chlamydomonas 2pac* mutant lacking the *RBD1* gene is specifically deficient in PSII accumulation (6). Our detection of CP47, CP43, D1, D2, PsbH, and PsbE protein synthesis in *2pac* and *2pac ftsh1-1* strains rules out the possibility that the major role of RBD1 is in enabling translation of any of these PSII subunits. The present immunochase experiments and the increased accumulation of PSII in the *2pac ftsh1-1* double mutant show that the low levels of PSII in *2pac* are due in major part to a posttranslational instability of PSII subunits that can be partly alleviated by specific inactivation of the thylakoid-localized FtsH protease (Figs. 1 and 2). These data are consistent with the results obtained with the cyanobacterial mutant lacking the RBD1 ortholog RubA (9), in which the *ΔrubA* mutant was found to synthesize PSII subunits at close to WT levels. RBD1 is also necessary for PSII accumulation in Arabidopsis (6, 10). Recently, a reduction in D1 synthesis was reported in the Arabidopsis *rbd1* mutant (10). This phenotype was hypothesized to be due to improper insertion of D1 into the membrane, which was proposed to be caused by the absence or unavailability of a cofactor.

### PSII that accumulates in *2pac ftsh1-1* is highly sensitive to light and possibly structurally distinct from WT PSII

We found that the *2pac ftsh1-1* strain was able to grow photoautotrophically (Fig. 4*A*) indicating that at least some amount of functional PSII was present since it was able to sustain growth. These results are consistent with the observation that the cyanobacterial Δ*rubA* mutant is able to grow photoautotrophically (6, 9). The activity of PSII from *2pac ftsh1-1* was the highest in dark-grown cells, as evidenced by the partial recovery of variable fluorescence (Fig. 5*A*).

**Figure 7:**
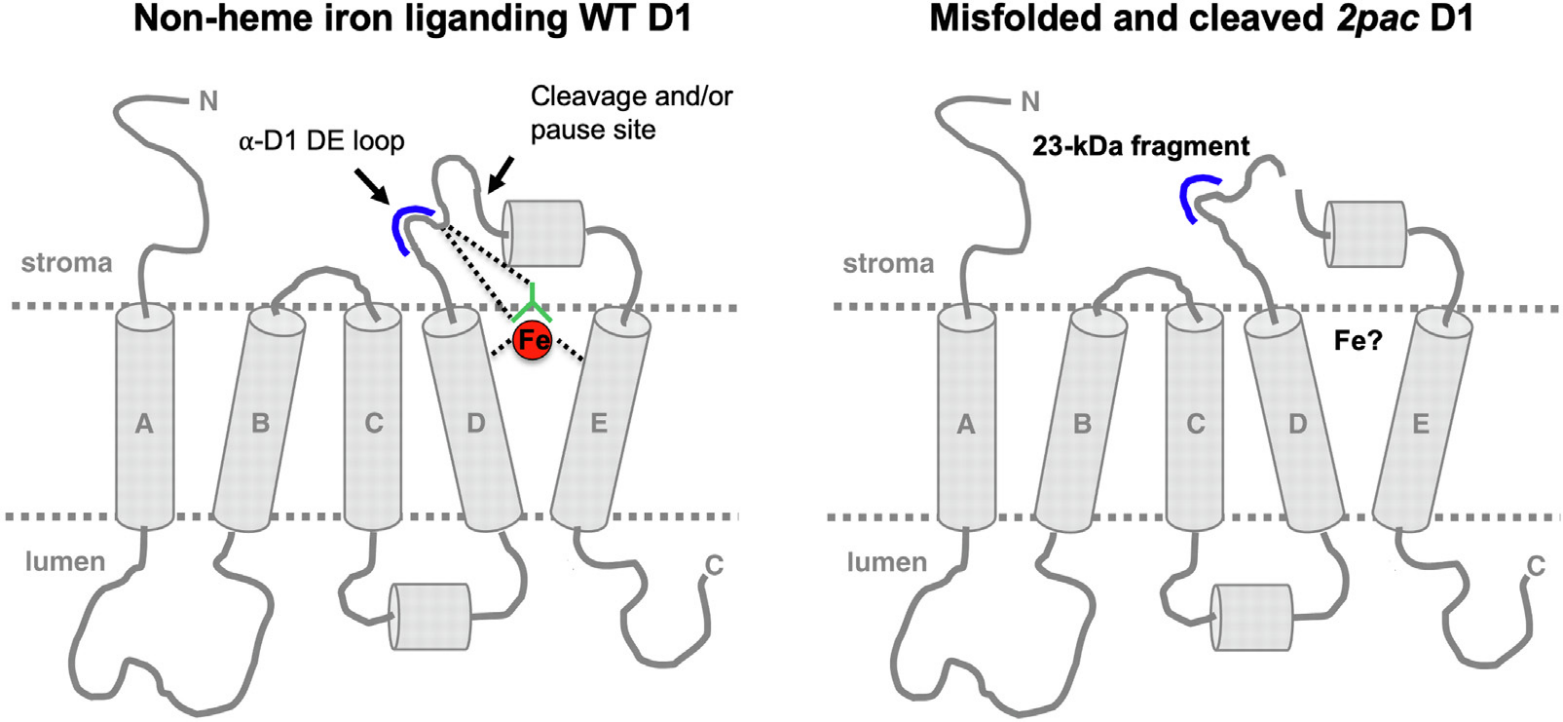
**Model showing the effect of RBD1 mutation on the folding/maturation of D1.** WT D1 (*left side*) provides four ligands that directly or indirectly (*via* bicarbonate, *green*) coordinate the nonheme iron of PSII (*red sphere*). These direct ligands are located in helix D (H215), helix E (H272), and in the DE-loop (E244 and Y246) close to the DE loop peptide (residues 234–242: NEGYRFGQE). A conformational change of the DE-loop of D1 may occur in the *2pac* mutant that lacks RBD1 (*right side*), resulting in proteolytic cleavage, possibly mediated by a Deg protease, followed by FtsH-mediated degradation. This conformational change may be due to the absence of the nonheme iron, which we propose may be delivered by RBD1 to D1 during the assembly of PSII (model adapted from (13, 63, 64)). PSII, photosystem II.

The D1 protein of PSII is the main target of photodamage (28). The accumulation of D1 fragments in the single mutant *ftsh1-1* is consistent with proposed models for plant (29, 30) and algal chloroplasts (13) in which there is a joint action between Deg proteases (endoproteolytic cuts) and FtsH proteases (processive degradation) during the repair of photodamaged PSII. Our detection of the 23-kDa fragment of D1 in dark-grown *2pac ftsh1-1* cells (Fig. 5*B*) is highly unusual and suggests that the conformation of PSII in the mutant is altered. This fragment has been hypothesized to be either a degradation intermediate (23) or a product of translational pausing (24). Reports presenting evidence that the fragment is due to degradation have hypothesized that it results from proteolysis (31, 32, 33) or direct damage by reactive oxygen species (34). In both cases, the trigger for degradation is thought to be photoinhibition, which would not occur in complete darkness. Indeed, the 23-kDa fragment is not detected in dark-grown *ftsh1-1* (13). However, if the trigger were instead a conformational change occurring as a result of photodamage or misfolding (as might be the case in the absence of RBD1), the observed data would support a model in which RBD1 is required for the proper folding of D1 during PSII assembly (Fig. 7). A conformational change has indeed been previously suggested to act as a signal recognized by proteases that catalyze cleavage (31, 32, 35). Specifically, occupation of the Q_B_ site by the PSII inhibitor PNO8 (*N*-octyl-3-nitro-2,4,6-trihydroxybenzamide) triggers the production of the 23-kDa fragment in darkness, a process hypothesized to be due to PNO8-induced conformational changes at the DE loop (Nakajima *et al*., 1996). The stromal Arabidopsis Deg2 and Deg7 proteases have been shown to generate a 23-kDa and ∼20-kDa fragment of D1, respectively, *in vitro* (36). Deg2 does not appear to have a role in D1 turnover after photoinhibition (37), but Deg7 participates in PSII repair in Arabidopsis *in vivo* (38). The *Chlamydomonas* genome encodes 14 predicted Deg proteins (39), three of which appear to be localized to the stroma. Based on sequence homology to two stroma-localized Arabidopsis proteins, Deg2 and Deg7 are likely found in the stroma (40, 41) while Deg1C, an ortholog of lumen-localized Arabidopsis Deg1, was experimentally identified in the stroma (42). Additionally, large-scale proteomic studies utilizing mass spectrometry have detected both Deg7 (43) and Deg1C (44). The sole study dedicated to a *Chlamydomonas* Deg mutant is the characterization of the *deg1C* mutant which accumulates proteins involved in high light acclimation when grown at low light intensities (42). It is possible that Deg2, Deg7, and/or Deg1C function in PSII assembly quality control when RBD1 is absent, leading to production of the 23-kDa fragment in darkness and its accumulation in the *2pac ftsh1-1* strain but not in *ftsh1-1*.

Alternatively, if the observed 23-kDa fragment is a translational pause product (24), then RBD1 might be required to facilitate the full translation of D1. This model, however, is not supported by the results of our pulse-labeling experiments in which we observed no differences in the rate of translation of full-length D1 in the *2pac* mutant (Fig. 1*A*), given our 7-min pulse-labeling time which favors the detection of the rate of translation rather than the rate of degradation. We would have expected to detect a 23-kDa fragment in the *2pac ftsh1-1* strain during the same pulse-labeling experiment if it was due to a pause in D1 synthesis, but no such band was observed (Fig. 1*A*). However, the experiment requires a pulse under light since the incorporation of acetate is light-dependent, so it is still possible that pausing occurs in the dark.

The presence of aberrant PSII complexes in dark-grown *2pac ftsh1-1* would be consistent with the proposed role of RBD1 and its cyanobacterial ortholog RubA in PSII assembly (8, 9), but not with its separate proposed function in protecting assembly intermediates from photooxidative damage (8). The role in PSII assembly is further supported by the accumulation of RC47 and a smaller PSII assembly subcomplex detected *via* 2D-PAGE of *2pac ftsh1-1* thylakoids (Fig. 3) and by the observed interaction of RubA with two such PSII assembly subcomplexes during PSII assembly in cyanobacteria (9).

### There are no detectable differences in protein composition of PSII from *2pac ftsh1-1* relative to the WT

The extreme light-sensitivity of PSII from *2pac ftsh1-1* compared to the WT led us to hypothesize that there might be a difference in protein and/or cofactor composition between the mutant and WT reaction centers. Mass spectrometry analysis of purified PSII complexes was unable to resolve any differences in the presence or absence of subunits (Table 1). This technique, however, suffers from the limitation that the detection of small hydrophobic proteins, of which there are many in PSII, is difficult. Consequently, we were unable to detect many of the small subunits of PSII, two of which (PsbJ and PsbL) are encoded by genes that lie directly downstream of *rubA* in the genomes of nearly all cyanobacteria (Calderon *et al*, 2013). It is therefore possible that one or more of these subunits is absent in the reaction centers purified from *2pac ftsh1-1*.

Our detection of cytochrome *b*_*559*_ in isolated PSII complexes (Fig. 6*C*) indicates that the *b-*heme coordinated by PsbE and PsbF (cytochrome *b*_559_) is unaltered and present in approximately WT levels in the *2pac ftsh1-1* mutant, demonstrating that RBD1 is unlikely to play a role in the maturation of cytochrome *b*_559_. While rubredoxins have generally been described as redox-active electron transport proteins (45), there is emerging evidence that rubredoxins may act as iron delivery proteins. Mechanical rupture experiments that enable monitoring of the folding and unfolding of rubredoxins around their iron centers have revealed that the two- and three-cysteine coordinated iron centers that exist during iron release are kinetically stable (46). More recently, the rubredoxin domain of 3-hydroxyanthranilate 3,4-dioxygenase was observed to directly transfer its bound iron to an empty nonheme iron–binding site from the same enzyme (47). RBD1 could conceivably serve a similar role for PSII, which binds a nonheme iron (Fig. 7). Both the nonheme iron of PSII and the iron-binding domain of RBD1/RubA are on the stromal side of the thylakoid membrane (8, 9, 48, 49). This nonheme iron is located at the electron acceptor side of PSII between the two quinones, Q_A_ and Q_B_. It is coordinated by four histidine residues, two from D1 and two from D2, and by a bicarbonate ion that provides a bidentate ligand (49, 50). Many amino acid residues in the DE loop of the D1 protein, which are also in the vicinity of the nonheme iron (F239, Q241, E242, Y246), have been previously reported to undergo oxidation (51), and the amino acids between 238 and 248 have been proposed to be the region where cleavage of the DE loop occurs *in vivo* (52), yielding the 23-kDa fragment. The 23-kDa fragment of D1 that we observe in dark-grown (and therefore non-photodamaged) *2pac ftsh1-1* is likely due to a conformational change at the DE loop, possibly triggered by the absence of the nonheme iron (or replacement with a different metal). Indeed, specific perturbations of the nonheme iron have been reported to affect cleavage of D1 and the appearance of the 23-kDa fragment (34).

A model in which RBD1 functions in iron delivery is consistent with the observation that the cyanobacterial RubA interacts with D1 during the formation of the D1-D2 heterodimeric complex RCII (9) but is not present in either of the direct precursors of RCII, the so-called D1_mod_ and D2_mod_ subcomplexes (53). The formation of the RCII complex is presumably accompanied by the insertion of the nonheme iron, given that both D1 and D2 provide the ligands for coordinating this metal (Fig. 7). Alternatively, RBD1/RubA could be involved in reduction of the nonheme iron (6) during its ligation to the D1-D2 heterodimer. Unfortunately, our attempts to detect the presence or absence of the nonheme iron of PSII *via* electron paramagnetic resonance and inductively coupled plasma-MS on isolated PSII from *2pac ftsh1-1* were unsuccessful. Nonetheless, the possibility that RBD1 delivers or reduces the nonheme iron during PSII assembly is compatible with all currently available data.

## Experimental procedures

### *Chlamydomonas* strains, mutant generation, and growth conditions

WT (4A+) and mutant strains *2pac* (6), Fud7 (15), *ftsh1-1* (13), H-HIS (27), and *2pac ftsh1-1* were grown at 25 °C on TAP medium (16) or high salt minimal medium (25) as indicated. Crosses were performed as described (54). Strains were grown at various light intensities from dark to 30 μmol photons m^−2^ s^−1^, as indicated in the figure legends. Mutants were genotyped based on resistance to paromomycin (for *2pac*) or by PCR (for *2pac* or *ftsh1-1*) as described (6, 13) or for H-HIS *via* immunoblotting with a monoclonal NOVAGEN HIS TAG antibody (Sigma-Aldrich).

### Generation of *2pac ftsh1-1* double mutant

The *2pac* (mating type +) and *ftsh1-1* (mating type -) mutants were crossed. Four full tetrads, nine triads, and one dyad of the resulting progeny were assayed for the presence/absence of the *ftsh1-1* mutation by PCR (Fig. S1*A*). To ensure the progeny were derived from successful mating, progeny were genotyped at the mating-type locus *via* PCR. All progeny groups showed a mixture of + and – mating types, indicating that they were derived from the cross (Fig. S1*B*). Progeny were grown on TAP plates containing paromomycin to select for strains bearing the *2pac* mutation (Fig. S1*C*). One particular strain, T3.4, that contained both mutations was selected for further characterization and is the strain identified as *2pac ftsh1-1* when only one strain is shown and not specified.

### [^14^C]-acetate pulse-labeling

Exponentially growing cells at 2 × 10^6^ cells mL^−1^ from a 200 ml culture grown in TAP medium at 20 μmol photons m^−2^ s^−1^ were harvested by centrifugation, washed with minimum MIN-Tris medium, and resuspended in 5 ml MIN-Tris medium at 2 × 10^7^ cell/ml. Cells were allowed to deplete their intracellular carbon pool for 1 h under 20 μmol photons m^−2^ s^−1^ and strongly agitated for a good aeration. Afterward, both the cytosolic translation inhibitor cycloheximide (final concentration 10 μg/ml) and [^14^C]-acetate (PerkinElmer, NEC084; final concentration 10 μCi mL^−1^) were added simultaneously. Cells were allowed to take up the radiolabeled acetate for 7 min at 20 μmol photons m^−2^ s^−1^. The pulse-labeling was stopped by adding 35 ml of ice-chilled TAP medium containing 50 mM nonradioactive acetate followed by centrifugation at 4 °C. Cells were resuspended in ice-chilled Hepes washing buffer, centrifuged, and immediately resuspended in ice-cold 0.2 M DTT and 0.2 M Na_2_CO_3_, frozen in liquid nitrogen and kept at −80 °C until analysis by SDS-PAGE. Whole-cell protein extracts were separated by SDS-PAGE on a 12%-18% polyacrylamide gel in the presence of 8 M urea and visualized by autography. The assignment of the bands is based on mutant analysis (5, 55).

### Immunochase

Inhibitors of chloroplast gene translation (chloramphenicol, 100 μg mL^-1^ and lincomycin, 500 μg mL^−1^) were added to 400 ml of exponentially growing cells in low light and TAP. Cells were incubated, with shaking, at 6 μmol photons m^−2^ s^−1^ over the course of 4 h, and 50 ml of cells were harvested at 0, 1, and 4 h postaddition of inhibitors before extraction of proteins, as described (6). Protein samples were separated by SDS-PAGE on 12 to 18% polyacrylamide gels containing 8 M urea before transfer *via* semi-dry transfer system and subsequent blotting with specific polyclonal antibodies against ATP synthase (CF_1_ α and β subunits, PSII reaction center subunits D1-DE loop (Agrisera AS10704) and D2 (Agrisera AS06146), PSII core antennae CP43 and PSII extrinsic subunit OEE3).

### Chlorophyll fluorescence measurements

Chlorophyll fluorescence kinetics were measured at room temperature on dark-adapted cells. A home-built fluorimeter with a green detecting light was used for measurements on 1-ml aliquots of liquid cultures (56) before and after the addition of PSII-specific inhibitor 3-(3,4-dichlorophenyl)-1,1-dimethylurea (DCMU; 10 μM). A fluorescence imaging system (BeamBio, SpeedZen camera) with a blue detecting light was used for measurements of plates as described (57). The maximum quantum yield of PSII photochemistry (F_v_/F_m_) was calculated as (F_m_-F_0_)/F_m_, where F_0_ is the fluorescence level of dark-adapted cells in the absence of DCMU and F_m_ is the maximum level of fluorescence in the presence of DCMU or after a saturating light pulse.

### Analysis of thylakoid membrane proteins and complexes

SDS-PAGE and immunoblot analysis of proteins were performed as previously described (6) with each lane containing protein corresponding to 8 μg total chlorophyll. Thylakoid membranes were isolated as described (58). Briefly, cells were harvested at logarithmic growth phase (2 × 10^6^ cells mL^−1^) and washed in MKT buffer (10 mM Tricine–KOH, pH 7.5, 20 mM KCl, 25 mM MgCl_2_, 5 mM aminocaproic acid, 1 mM benzamidine, and 0.2 mM PMSF) once before breaking by passage through a French pressure cell. Membranes were collected by centrifugation at 31,000*g* for 30 min, then resuspended in ACA 750 (750 mM aminocaproic acid, 50 mM Bis–Tris, pH 7.0, 0.5 mM EDTA) to a concentration of 1 mg mL^−1^ chlorophyll. Membranes were solubilized by addition of an equal volume of aminocaproic acid 750 containing 2% n-dodecyl β-D-maltoside (β-DM, Anatrace) for a final concentration of 0.5 mg ml^-1^ chlorophyll and 1% β-DM. Membranes were solubilized for 10 min on ice in the dark before centrifugation to pellet unsolubilized material. Solubilized membranes were then mixed 60:1 with loading buffer (100 mM BisTris–HCl, pH 7.0, 5% Coomassie G-250, 0.5 mM aminocaproic acid, and 30% sucrose) and 15 μl (corresponding to 7.5 μg chlorophyll) were loaded onto a 4 to 16% precast BN-PAGE gel (Life Technologies). Second dimension analysis was performed by solubilizing BN-PAGE gel slices in 2× Laemmli buffer (59) and loading into precast 2D gels (Life Technologies). All 2D images were taken on an AlphaInnotech Alphaimager using identical exposure times of 30 s.

Mass spectrometry experiments were performed at the Vincent J. Coates Protein Mass Spectrometry Facility. Bands were excised from BN-PAGE gels, digested with trypsin, and subjected to analysis by reverse phase LC-MS/MS on a Thermo Scientific LTQ XL ion trap mass spectrometer.

### Purification of HIS-tagged PSII

PSII purification was performed as described (27), with the following minor modifications. Cultures were grown in 10 l bottles with vigorous stirring and bubbled with filtered air. All purification steps were performed in the dark, and all buffers were supplemented with betaine to a final concentration of 1 M. To account for differences in protein/chlorophyll content in the *2pac ftsh1-1* mutant, membranes from *2pac ftsh1-1* (T7A) were solubilized at a final concentration of 0.6 mg mL^−1^ chlorophyll and 25 mM β-DM. Samples were further purified before analysis by MS by loading directly onto a precast 4 to 16% BN-PAGE gel (Life Technologies).

### Quantification of heme *b*_559_ levels

UV-visible spectra of purified PSII samples were obtained on a SPECTRAmax *PLUS* spectrophotometer (Molecular Devices) under fully-reduced or fully-oxidized conditions after saturating treatment with dithionite or ferricyanide, respectively. Extinction coefficients for heme *b*_559_ (60) and chlorophyll (61) were used to calculate the concentrations of these molecules in the samples.

## Data availability

All data are contained within the article.

## Supporting information

This article contains supporting information.

## Conflict of interest

K. K. N. is an investigator of the Howard Hughes Medical Institute. The authors declare that they have no conflicts of interest with the contents of this article.

## Figures and Tables

**Figure:**
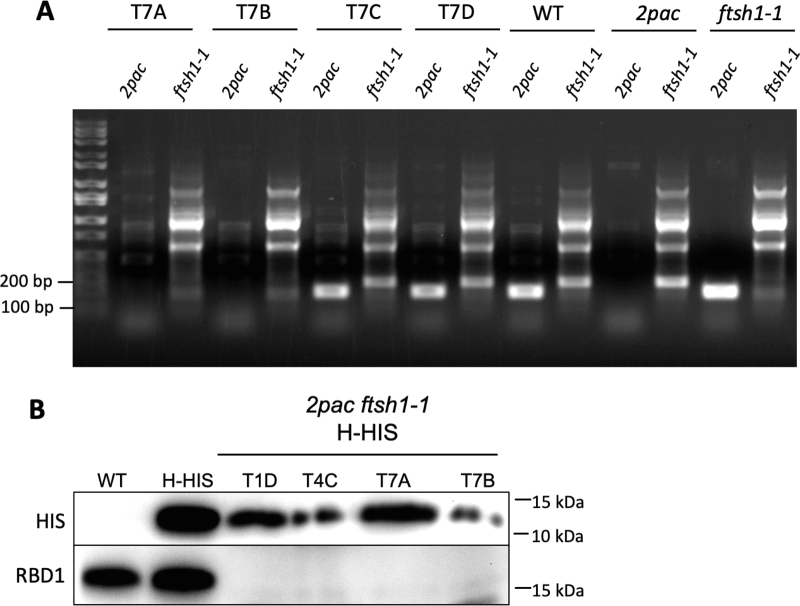
Figure S5Isolation of *2pac ftsh1-1* double mutants in the H-HIS background. *A*, PCR analysis of *FTSH1* locus and *2pac* allele from a representative tetrad. Single band in “*ftsh1-1”* lane corresponds to wild-type *FTSH1* allele, while three diffuse bands corresponds to *ftsh1-1* mutation. Single band in “*2pac*” lane corresponds to the wild-type *RBD1* allele, while absence corresponds to the *2pac* deletion. *B*, immunoblot confirmation of the presence of H-HIS (*via* α-HIS immunodetection) and absence of RBD1 (*via* α-RBD1 immunodetection) in selected *2pac ftsh1-1* mutants isolated from the cross with H-HIS. Strain T7A was used for the experiments in Fig. 6, Tables 1, S1 and Fig. S6.
