# Supplementary material for: Rubredoxin 1 promotes the proper folding of D1 and is not required for heme b559 assembly in Chlamydomonas photosystem II
Source: J Biol Chem. 2023 Feb 2;299(3):102968. doi: 10.1016/j.jbc.2023.102968 (PMC9986647; doi:10.1016/j.jbc.2023.102968)
Supplement: Table S1 [file mmc1.docx]

**Table S1: PSII proteins and peptides identified via LC-MS/MS analysis of isolated PSII dimers from H-HIS and H-HIS *2pac ftsh1-1* (T7A)**

| **Protein name** | **Uniprot  No.** | **Gene** | **Protein  MW (kD)** | **Peptide  MH+ (D)** | **Peptide Sequence** | **H-HIS spectral  counts** | **T7A spectral  counts** |  |
| --- | --- | --- | --- | --- | --- | --- | --- | --- |
| D1 | P07753 | *psbA* | 39 | 1119.2 | R.RENSSLWAR.F | 59 | 13 |  |
|  |  |  |  | 962.5 | R.ENSSLWAR.F | 8 | 21 |  |
|  |  |  |  | 1444.2 | R.FCEWITSTENR.L | 6 | 23 |  |
|  |  |  |  | 966.5 | R.EWELSFR.L | 2 | 13 |  |
|  |  |  |  | 1500.3 | R.ETTENESANEGYR.F | 6 | 6 |  |
|  |  |  |  | 2189.4 | R.FGQEEETYNIVAAHGYFGR.L | 2 | 10 |  |
|  |  |  |  | 1459.9 | R.LIFQYASFNNSR.S | 14 | 15 |  |
|  |  |  |  | 1314.7 | R.VLNTWADIINR.A | 94 | 229 |  |
|  |  |  |  | 1288.3 | R.ANLGMEVMHER.N | 10 | 33 |  |
|  |  |  |  | 1303.2 | R.ANLGMoxEVMHER.N | 2 | 9 | # |
|  |  |  |  | 1303.2 | R.ANLGMEVMoxHER.N | 13 | 17 | # |
|  |  |  |  | 1319.2 | R.ANLGMoxEVMoxHER.N | 4 | 9 | # |
|  |  |  |  |  | TOTAL | **220** | **398** |  |
|  |  |  |  |  |  |  |  |  |
| D2 | P06007 | *psbD* | 39 | 1441.2 | R.TWFDDADDWLR.Q | 7 | 41 |  |
|  |  |  |  | 2031.3 | R.AFNPTQAEETYSMVTANR.F | 11 | 22 |  |
|  |  |  |  | 2046.6 | R.AFNPTQAEETYSMoxVTANR.F | 16 | 13 | # |
|  |  |  |  | 1227.8 | R.AYDFVSQEIR.A | 48 | 19 |  |
|  |  |  |  | 1547.8 | R.AAEDPEFETFYTK.N | 5 | 31 |  |
|  |  |  |  | 1041.7 | K.NILLNEGIR.A | 71 | 67 |  |
|  |  |  |  | 1440.5 | R.AWMAAQDQPHER.L | 13 | 29 |  |
|  |  |  |  | 1455.7 | R.AWMoxAAQDQPHER.L | 20 | 23 | # |
|  |  |  |  | 1198.8 | R.LVFPEEVLPR.G | 85 | 181 |  |
|  |  |  |  |  | TOTAL | **276** | **426** |  |
|  |  |  |  |  |  |  |  |  |
| cytochrome *b*_559_ alpha | P48268 | *psbE* | 9 | 1149.8 | R.PFSDILTSIR.Y | 18 | 16 |  |
|  |  |  |  | 1170.6 | R.PNEYFTEDR.Q | 7 | 6 |  |
|  |  |  |  | 1042.6 | R.QEAPLITDR.F | 2 | 5 |  |
|  |  |  |  | 1973.1 | R.QEAPLITDRFNALEQVK.K | 0 | 1 |  |
|  |  |  |  | 949.6 | R.FNALEQVK.K | 37 | 29 |  |
|  |  |  |  | 1077.4 | R.FNALEQVKK.L | 27 | 13 |  |
|  |  |  |  |  | TOTAL | **91** | **70** |  |
|  |  |  |  |  |  |  |  |  |
| cytochrome *b*_559_ beta | Q08363 | *psbF* | 5 | 1494.0 | K.SAEVLVYPIFTVR.W | 19 | 24 |  |
|  |  |  |  |  | TOTAL | **19** | **24** |  |
|  |  |  |  |  |  |  |  |  |
| CP43 | P56778 | *psbC* | 51 | 1726.6 | R.DQETTGFAWWSGNAR.L | 3 | 10 |  |
|  |  |  |  | 744.5 | R.LINLSGK.L | 20 | 203 |  |
|  |  |  |  | 2019.4 | K.AMYFGGVYDTWAPGGGDVR.V | 3 | 30 |  |
|  |  |  |  | 2036.4 | K.AMoxYFGGVYDTWAPGGGDVR.V | 11 | 30 | # |
|  |  |  |  | 1924.0 | R.VITNPTTNAAVIFGYLVK.S | 0 | 3 |  |
|  |  |  |  | 1443.1 | R.LGANVASAQGPTGLGK.Y | 112 | 66 |  |
|  |  |  |  | 1494.9 | R.SPTGEIIFGGETMR.F | 22 | 51 |  |
|  |  |  |  | 1511.8 | R.SPTGEIIFGGETMoxR.F | 34 | 56 | # |
|  |  |  |  | 2264.1 | R.SPTGEIIFGGETMoxRFWDFR.G | 1 | 3 | # |
|  |  |  |  | 968.6 | R.GPWLEPLR.G | 60 | 336 |  |
|  |  |  |  | 1878.9 | R.GPWLEPLRGPNGLDLNK.L | 4 | 11 |  |
|  |  |  |  | 927.6 | R.GPNGLDLNK.L | 30 | 8 |  |
|  |  |  |  | 1429.4 | K.LKNDIQPWQER.R | 94 | 35 |  |
|  |  |  |  | 1187.0 | K.NDIQPWQER.R | 4 | 4 |  |
|  |  |  |  | 764.6 | R.AAAAGFEK.G | 3 | 3 |  |
|  |  |  |  | 1535.0 | K.GIDRFDEPVLSMR.P | 8 | 7 |  |
|  |  |  |  | 1551.8 | K.GIDRFDEPVLSMoxR.P | 0 | 15 | # |
|  |  |  |  | 1860.7 | K.GIDRFDEPVLSMRPLD.- | 6 | 5 |  |
|  |  |  |  | 1876.5 | K.GIDRFDEPVLSMoxRPLD.- | 40 | 111 | # |
|  |  |  |  | 1093.4 | R.FDEPVLSMR.P | 7 | 4 |  |
|  |  |  |  | 1109.6 | R.FDEPVLSMoxR.P | 6 | 2 | # |
|  |  |  |  | 1419.3 | R.FDEPVLSMRPLD.- | 17 | 31 |  |
|  |  |  |  | 1435.3 | R.FDEPVLSMoxRPLD.- | 21 | 19 | # |
|  |  |  |  |  | TOTAL | **506** | **1043** |  |
|  |  |  |  |  |  |  |  |  |
| CP47 | P37255 | *psbB* | 56 | 1207.4 | R.VHTVVINDPGR.L | 84 | 42 |  |
|  |  |  |  | 1343.0 | R.QGMFVLPFMoxTR.L | 0 | 2 | # |
|  |  |  |  | 1343.4 | R.QGMoxFVLPFMTR.L | 0 | 1 | # |
|  |  |  |  | 1361.3 | R.QGMoxFVLPFMoxTR.L | 2 | 4 | # |
|  |  |  |  | 757.6 | K.TALDLPK.I | 19 | 20 |  |
|  |  |  |  | 1846.2 | R.YQWDQGFFQQEIQK.R | 22 | 103 |  |
|  |  |  |  | 1748.8 | R.VQASLAEGASLSDAWSR.I | 82 | 163 |  |
|  |  |  |  | 1952.5 | R.IPEKLAFYDYIGNNPAK.G | 1 | 1 |  |
|  |  |  |  | 1486.7 | K.LAFYDYIGNNPAK.G | 112 | 135 |  |
|  |  |  |  | 2094.0 | R.TGAMoxNSGDGIAVGWLGHASFK.D | 0 | 1 | # |
|  |  |  |  | 3152.6 | R.MPTFFETFPVLLLDKDGIVRADVPFRK.A | 1 | 0 |  |
|  |  |  |  | 704.7 | R.ADVPFR.K | 3 | 2 |  |
|  |  |  |  | 833.3 | R.ADVPFRK.A | 0 | 16 |  |
|  |  |  |  | 1452.7 | R.KAQLGEIFEFDR.S | 0 | 1 |  |
|  |  |  |  | 1324.9 | K.AQLGEIFEFDR.S | 18 | 49 |  |
|  |  |  |  | 1109.5 | R.STLQSDGVFR.S | 28 | 26 |  |
|  |  |  |  | 1998.5 | R.DVFAGIDDDINDQVEFGK.Y | 18 | 30 |  |
|  |  |  |  | 976.9 | K.KLGDTSSLR.E | 29 | 43 |  |
|  |  |  |  | 848.6 | K.LGDTSSLR.E | 8 | 20 |  |
|  |  |  |  |  | TOTAL | **427** | **659** |  |
|  |  |  |  |  |  |  |  |  |
| PSII H subunit | P22666 | *psbH* | 9 | 692.9 | -.MATGTSK.A | 1 | 0 |  |
|  |  |  |  | 2058.0 | K.VNSDFQEPGLVTPLGTLLR.P | 26 | 49 |  |
|  |  |  |  | 2855.1 | K.VNSDFQEPGLVTPLGTLLRPLNSEAGK.V | 0 | 2 |  |
|  |  |  |  | 815.7 | R.PLNSEAGK.V | 4 | 2 |  |
|  |  |  |  |  | TOTAL | **31** | **53** |  |
|  |  |  |  |  |  |  |  |  |
| PSII OEE1 | P12853 | *psbO* | 31 | 1450.2 | R.LTYTLDAMoxSGSFK.V | 1 | 1 | # |
|  |  |  |  | 1450.2 | R.GGSTGYDNAVALPAR.A | 2 | 2 |  |
|  |  |  |  | 1919.3 | K.GSGIANTCPVLESGTTNLK.E | 0 | 2 |  |
|  |  |  |  |  | TOTAL | **3** | **5** |  |
| # = oxidized methionine | |  |  |  |  |  |  |  |
| Peptide detected only in H-HIS | | |  |  |  |  |  |  |
| Peptide detected only in T7A | | |  |  |  |  |  |  |
